# Editorial: Rational drug design of metal complexes for cancer therapy

**DOI:** 10.3389/fchem.2022.1035274

**Published:** 2022-09-20

**Authors:** Tânia S. Morais, Alexandra R. Fernandes, Pedro V. Baptista, Dinorah Gambino

**Affiliations:** ^1^ Centro de Química Estrutural, Institute of Molecular Sciences, Faculdade de Ciências, Universidade de Lisboa, Lisboa, Portugal; ^2^ i4HB-UCIBIO, Departamento de Ciências da Vida, Faculdade de Ciências e Tecnologia, Universidade Nova de Lisboa, Caparica, Portugal; ^3^ Área Química Inorgánica, Facultad de Química, Universidad de la República, Montevideo, Uruguay

**Keywords:** cancer chemotherapy, inorganic/organometallic, medicinal chemistry, metal-based drug design, drug delivery, mechanism of action, nanomedicine

The growing burden of cancer incidence and mortality is one of the most important public health problems with a strong socio-economic impact worldwide. Despite decades of global research efforts, there is still no cure for cancer due to the absence of efficient and specific therapies, which results in high recurrence and relapse rates, thus perpetuating the leading role of cancer in global mortality (Debela et al., 2021). In this context, novel effective anticancer therapies capable of reducing the side effects caused by conventional treatments is one of the biggest challenges towards overcoming the high incidence and prevalence of the disease. In the last years, metal-based anticancer drugs have gained an undisputed role in cancer therapy, as evidenced by the clinical success of platinum-based anticancer drugs. In fact, it is estimated that, in the last decade, more than half of patients receiving anticancer chemotherapy were treated with therapeutic regimens containing platinum complexes (Johnstone et al., 2014). Despite some limitations, such as severe toxic side effects and acquired resistance, their evident clinical success has inspired a growing number of researchers to develop new therapeutic approaches based on metal complexes (Ndagi et al., 2017). These efforts have seen significant advances due to the input of the rational design of metal compounds for cancer treatment, but the high occurrence of multidrug resistance and severe side effects associated with chemotherapy still remain a challenge. As such, new generations of metal compounds with new mechanisms of action, broader spectra and improved anticancer properties are still needed.

The Research Topic “Rational Drug Design of Metal Complexes for Cancer Therapy” highlights several recent developments in the field of metal compounds for cancer therapy. The issue comprises 5 selected peer-reviewed manuscripts (three original research articles and two reviews) covering research on design and synthesis, structure-activity relationships, mechanistic studies regarding biological targets and pathways involved, as well as important applications and recent developments in effect of metal complex in the modulation of the metabolic pathways of cancer. Green et al. report the synthesis and the antiproliferative activity om HCT116, SW480, SiHa and NCI-H460 cells of a series of Os(III)-Ru(II), Rh(III)-Ru(II), Os(II)-Rh(III), Ir(III)-Rh(III), Ir(III)-Ru(II) and Ir(III)-Os(II) heterodimetallic complexes based on a ditopic ligand featuring 2-pyridylimine chelating motifs and organometallic half-sandwich moieties. An innovative combination of approaches is proposed by Castro and co-workers, who evaluate the efficacy of thiopyridinium Zn(II) phthalocyanines (ZnPcs) conjugates for photodynamic therapy (PDT) B16F10 melanoma cells. In this article, the authors describe the photophysical, photochemical, and *in vitro* photobiological properties of ZnPcs and correlate the number of charge units, and the presence/absence of a-F atoms on the phthalocyanine structure with the PDT efficacy of the complexes. Focusing on the combinatory effect of strategies, Meier-Menches et al. explored the profile of cellular responses and characterized the differentiation–and metal-specific effects of the clinically used combination treatment arsenic trioxide (ATO) and all-trans retinoic acid (ATRA) in acute myeloid leukemia (AML) cells. The authors also investigate the response of the organoruthenium compound plecstatin-1 in combination with the ATRA. An excellent review by HYPERLINK Kou et al. discusses the recent developments on gold complexes as modulators of tumor cell metabolism. The authors discuss the rationale underlying the anti-tumor effects of the gold compounds based on their effects on glucose, protein, and nucleic acid metabolism. Finally, Murillo et al. outline the applications of transition metal complexes as anticancer agents acting through changes in the intracellular redox balance and interaction with redox enzymes.

The editors hope that this research topic will contribute with new and useful information to the progress of research and development of new approaches based on metal complexes for cancer therapy.

## References

[B1] Debela D. T. Muzazu S. G. Heraro K. D. Ndalama M. T. Mesele B. W. Haile D. C. (2021). New approaches and procedures for cancer treatment: Current perspectives. SAGE open Med. 9, 205031212110343. 10.1177/20503121211034366 PMC8366192 34408877

[B2] Johnstone T. C. Park G. Y. Lippard S. J. (2014). Understanding and improving platinum anticancer drugs – phenanthriplatin. Anticancer Res. 34, 471. 24403503 PMC3937549

[B3] Ndagi U. Mhlongo N. Soliman M. E. (2017). Metal complexes in cancer therapy - an update from drug design perspective. Drug Des. devel. Ther. 11, 599–616. 10.2147/DDDT.S119488 PMC5344412 28424538

